
JOURNAL INFORMATION
==============================
NLM Title Abbreviation: Biospektrum (Heidelb)
Journal ID: Biospektrum (Heidelb)
NLM Journal ID: 9615685

Biospektrum

ISSN: 0947-0867
EISSN: 1868-6249

ARTICLE INFORMATION
==============================
PMCID: PMC8111368
PMID: 33994671
DOI: 10.1007/s12268-021-1586-4
Article ID: 1586
Article version: 1
Subjects: Editorial

Deutsche COVID-19 Omics Initiative (DeCOI)

Schultze Joachim L. joachim.schultze@dzne.de
1 2
1 grid.424247.3 0000 0004 0438 0426 Direktor Systemmedizin und PRECISE Platform for Single Cell Genomics and Epigenomics, Deutsches Zentrum für Neurodegenerative Erkrankungen e.V. (DZNE), Venusberg-Campus 1/99, D-53127 Bonn, Deutschland
2 grid.10388.32 0000 0001 2240 3300 Genomik & Immunregulation, Life & Medical Sciences (LIMES) Institut, Universität Bonn, Bonn, Deutschland
Electronic publication date: 2021 May 11
Print publication date: 2021
Volume: 27
Issue: 3
First page: 227
Last page: 227
Copyright: © Der Autor 2021
License: Dieser Artikel wird unter der Creative Commons Namensnennung 4.0 International Lizenz veröffentlicht, welche die Nutzung, Vervielfältigung, Bearbeitung, Verbreitung und Wiedergabe in jeglichem Medium und Format erlaubt, sofern Sie den/die ursprünglichen Autor(en) und die Quelle ordnungsgemäß nennen, einen Link zur Creative Commons Lizenz beifügen und angeben, ob Änderungen vorgenommen wurden. Die in diesem Artikel enthaltenen Bilder und sonstiges Drittmaterial unterliegen ebenfalls der genannten Creative Commons Lizenz, sofern sich aus der Abbildungslegende nichts anderes ergibt. Sofern das betreffende Material nicht unter der genannten Creative Commons Lizenz steht und die betreffende Handlung nicht nach gesetzlichen Vorschriften erlaubt ist, ist für die oben aufgeführten Weiterverwendungen des Materials die Einwilligung des jeweiligen Rechteinhabers einzuholen. Weitere Details zur Lizenz entnehmen Sie bitte der Lizenzinformation auf http://creativecommons.org/licenses/by/4.0/deed.de.
License URL: https://creativecommons.org/licenses/by/4.0/

issue-copyright-statement: © Springer-Verlag GmbH Deutschland, ein Teil von Springer Nature 2021

==============================
Danksagung

Ich bedanke mich bei allen Mitgliedern von DeCOI für die exzellente Zusammenarbeit, die vielen gemeinsamen Stunden, die wir gemeinsam mit Wissenschaft verbracht haben, die Offenheit und der unerschütterliche Wille, gemeinsam einen kleinen Beitrag zur Bewältigung der Krise zu leisten.

■ Nach über einem Jahr Pandemie wissen wir, wie wichtig wissenschaftlicher Erkenntnisgewinn für die Krisenbewältigung ist. Für die Bevölkerung ist dies am Beispiel der schnellen Erforschung der Impfstoffe am leichtesten zu begreifen. Aber andere Wissenschaftsbereiche haben einen nicht minder wichtigen Anteil an der Krisenbewältigung. Hier kommt der Genomforschung eine Schlüsselrolle zu. Ohne Sequenzierung keine Entschlüsselung der Erbinformation des Virus. Ohne Erbinformation keine Impfung und keine molekulargenetische Surveillance zur Identifikation und Verbreitung gefährlicher Mutanten. Ohne Sequenzierung kein Hinweis auf Mutationen in für die Virusabwehr essenziellen menschlichen Genen.

In einer Bottom-Up-Bewegung trieben insbesondere die Mitglieder des German Human Genome Archive (GHGA) Konsortiums und des Netzwerkes der DFG-geförderten NGS-Kompetenzzentren (NGS-CN) am Anfang die Aktivitäten voran. Mitte März 2020 baten die NGS-CNs die DFG um Unterstützung nachdem die Universitäten der NGS-CNs in Kiel, Dresden, Tübingen, Saarbrücken, Bonn, Köln und Düsseldorf bereits der Öffnung der Sequenzier-Einrichtungen im Lockdown zugestimmt hatten. Am 30. März gab auch die DFG grünes Licht. Bereits wenige Tage zuvor hatten sich Mitglieder aus den NGS-CN, GHGA und vieler anderer Institute in Deutschland zur Deutschen COVID-19 OMICS Initiative (DeCOI, https://decoi.eu) zusammengeschlossen.

Im Sinne des schnellstmöglichen Erkenntnisgewinns war es essenziell, jegliche für den normalen Wissenschaftsbetrieb übliche Kompetition zwischen den Arbeitsgruppen durch das Prinzip des Teilens von Wissen, Expertise, SOPs, Analyse-Pipelines, Ethik-Dokumenten, Proben, Daten und Analyse-Kapazitäten zu ersetzen. Diese Team-orientierte Vorgehensweise hat DeCOI die entsprechende Geschwindigkeit ermöglicht und hat damit auch im internationalen Vergleich zeigen können, zu welchen Höchstleistungen Wissenschaftler an deutschen Institutionen gemeinsam in der Lage sind. Heute gehören DeCOI über 100 Wissenschaftler an mehr als 50 Institutionen in ganz Deutschland an. Interessierte Wissenschaftler sind herzlich eingeladen, bei DeCOI mitzuwirken.

Die Errungenschaften von DeCOI erstrecken sich auf weitere zukunftsträchtige Forschungsbereiche. So leisten DeCOI-Mitglieder in internationalen Konsortien zur genetischen Suszeptibilität bei schweren COVID-19-Erkrankungen einen wichtigen Beitrag. Aber auch die erstmalige Anwendung völlig neuer KI-Konzepte, wie das Swarm Learning zur Identifikation von COVID-19-Patienten mittels Blut-Transkriptom-Analysen, war nur durch den unermüdlichen Einsatz vieler DeCOI-Mitglieder überhaupt möglich [6].

Mit DeCOI hat die Genomforschung gezeigt, welche außerordentliche Stellung sie in der Krisenabwehr einnehmen kann. Es ist an der Zeit, dass die Ministerien ernsthaft über föderierte, institutionalisierte Strukturen für die deutsche Genomforschung nachdenken und zu deren Umsetzung ihren Beitrag leisten. Die Genomforscher in Deutschland sind bereit dazu.

Lesen Sie mehr zu DeCOI in der nächsten Ausgabe von BIOspektrum.


Funding note

Open Access funding enabled and organized by Projekt DEAL.


Literatur

[1] van der Toorn W, Oh D-Y, von Kleist M, working group on SARS-CoV-2 Diagnostics at RKI (2021) COVIDStrategyCalculator: A software to assess testing- and quarantine strategies for incoming travelers, contact person management and de-isolation. Patterns (N Y) 10026410.1016/j.patter.2021.100264 PMC8057763 33899035
[2] van der Toorn W, Oh D-Y, Bourquain D et al. (2021) An intra-host SARS-CoV-2 dynamics model to assess testing and quarantine strategies for incoming travelers, contact person management and de-isolation. Patterns (N Y) 10026210.1016/j.patter.2021.100262 PMC8057735 33899034
[3] Schulte-Schrepping J Reusch N Paclik D Severe COVID-19 Is Marked by a Dysregulated Myeloid Cell Compartment Cell 2020 182 1419 1440 10.1016/j.cell.2020.08.001 32810438 PMC7405822
[4] Aschenbrenner AC Mouktaroudi M Krämer B Disease severity-specific neutrophil signatures in blood transcriptomes stratify COVID-19 patients Genome Med 2021 13 7 10.1186/s13073-020-00823-5 33441124 PMC7805430
[5] Bernardes JP Mishra N Tran F Longitudinal Multi-omics Analyses Identify Responses of Megakaryocytes, Erythroid Cells, and Plasmablasts as Hallmarks of Severe COVID-19 Immunity 2020 53 1296 1314 10.1016/j.immuni.2020.11.017 33296687 PMC7689306
[6] Warnat-Herresthal S, Schultze H, Shastry KL et al. (2020) Swarm Learning as a privacy-preserving machine learning approach for disease classification. BioRxiv
